# Characteristics of nonsuicidal self-injury associated with suicidal ideation: evidence from a clinical sample of youth

**DOI:** 10.1186/s13034-015-0053-8

**Published:** 2015-07-08

**Authors:** Sarah E. Victor, Denise Styer, Jason J. Washburn

**Affiliations:** Department of Psychology, University of British Columbia, 2136 West Mall, Vancouver, BC V6T1Z4 Canada; Alexian Brothers Behavioral Health Hospital, 1650 Moon Lake Boulevard, Hoffman Estates, IL 60169 USA; Northwestern University Feinberg School of Medicine, Abbott Hall Suite 1204, 710 N Lake Shore Drive, Chicago, IL 60611 USA

**Keywords:** Nonsuicidal self-injury, Self-mutilation, Deliberate self-harm, Suicide, Suicidal ideation, Risk assessment

## Abstract

**Background:**

Nonsuicidal self-injury (NSSI) and suicidal ideation (SI) are both distressing and quite common, particularly in youth. Given the relationship between these two phenomena, it is crucial to learn how we can use information about NSSI to understand who is at greatest risk of suicidal thoughts. In this study, we investigated how characteristics of nonsuicidal self-injury related to SI among treatment-seeking adolescents and young adults.

**Methods:**

Data were collected during routine program evaluation for a self-injury treatment program. Correlations between recent SI and NSSI characteristics were calculated for adolescent and young adult patients (*N* = 1502).

**Results:**

Low severity methods of NSSI (e.g. banging) were more strongly associated with SI than high severity methods (e.g. breaking bones). SI was associated with intrapersonal (automatic) NSSI functions. SI was associated with some indices of NSSI severity, such as number of methods and urge for NSSI, but not with others, such as age of onset.

**Conclusions:**

This study provides a valuable opportunity to expand our knowledge of suicide risk factors beyond those that may apply broadly to self-injurers and to non-injurers (e.g., depression, substance use) to NSSI-related factors that might be specifically predictive of suicidal thoughts among self-injurers. Findings inform clinical risk assessment of self-injurious youth, a population at high risk of suicidal thoughts and behaviors, and provide further insight into the complex NSSI/suicide relationship.

## Background

Nonsuicidal self-injury (NSSI) is the intentional, self-directed destruction of bodily tissue engaged in for purposes neither suicidal nor socially sanctioned, and includes behaviors such as cutting, burning, or hitting [1]. NSSI is common among community populations of adolescents and young adults, with approximately 13 % of young adults [2] and 16-18 % of adolescents [3] reporting at least one incidence of NSSI in their lifetimes. NSSI is even more common among adolescent psychiatric patients, where rates can reach up to 80 % [4]. Engaging in NSSI has been associated with a variety of types of psychopathology, including depression [5], personality disorders [6], substance use [7], and disordered eating [8].

While NSSI is, by nature, not suicidal, it is common for individuals who engage in NSSI to have suicidal thoughts and behaviors. Among adolescents, several studies have demonstrated rates of suicidal ideation (SI) at least double that of non-injurers. These findings have been replicated cross-nationally in the US [9], China [10], and Sweden [11]; in all cases, the relationship remained even after removing individuals who had attempted suicide in addition to engaging in NSSI. A longitudinal study with high school students show that a history of NSSI was the strongest predictor of subsequent SI, surpassing other baseline measures of depression, SI, suicidal threat/gesture, or suicide attempt [12]. Among depressed adolescents being treated with antidepressant medications, NSSI was more strongly associated with SI than a history of attempted suicide [13] or other known risk factors for SI, including depression and hopelessness [14].

While the association of NSSI and SI has been well supported (see [15] for a comprehensive review, and [16] for a recent analysis of the co-occurrence of NSSI and SI longitudinally), less research has focused on the characteristics of NSSI that are most associated with SI. Self-injurers are a heterogeneous group, differing in the methods, frequency, and functions of their self-injurious behaviors [17, 18]. Given this heterogeneity in the population of people who self-injure, and the high prevalence of NSSI among youth, research that facilitates understanding which individuals are at highest risk of SI is crucial for identifying the youth most in need of intervention.

A number of studies have focused on how NSSI frequency and number of methods are associated with suicide attempts (see [19] for review); however, to date, little research has specifically investigated how NSSI characteristics are associated with SI. One notable exception is the recent work by Paul and colleagues in a sample of university students, showing that SI was associated with lifetime NSSI frequency in a curvilinear fashion; using NSSI for the functions “to help me cry” and “hope someone would notice something is wrong” were also associated with SI [20]. Although a welcome addition to the literature, this study has limited clinical generalizability because it used a sample of undergraduate college students, assessed only lifetime SI, and only examined lifetime frequency of NSSI and NSSI functions as potential correlates of SI.

In order to better characterize the risk of SI associated with NSSI in clinical populations, we conducted exploratory analyses examining SI in a clinical sample of individuals seeking treatment for self-injurious behavior. This sample included a large number of individuals seeking treatment for NSSI who are diverse in age, gender, and ethnic background, as well as in characteristics of NSSI, including frequency, methods, functions, urges, and clinical levels of NSSI, as defined by proposed diagnostic criteria.

## Methods

### Procedures

Archival data were collected from clinical outcome databases at a large, privately run hospital providing inpatient, partial hospitalization, and intensive outpatient treatment for a variety of mental disorders in children, adolescents, adults, and geriatric populations. For this study, data were drawn from adolescents (ages 11 to 17) and young adults (ages 18 to 25) receiving treatment in the Center for Self-Injury Recovery Services Program (SIRS), an acute care (inpatient, partial hospitalization, and intensive outpatient) treatment program specifically designed to treat self-injurious behavior. Enrollment in SIRS requires that self-injury, either nonsuicidal or suicidal, be the patient’s primary presenting problems; patients could also have secondary diagnoses, such as eating disorders, mood disorders, or substance use disorders.

As part of routine clinical assessment and program evaluation, patients completed a detailed assessment of their NSSI at intake to, and discharge from, treatment. Patients were also assigned up to five diagnoses based on ICD-9 diagnostic criteria by an attending psychiatrist using a non-standardized clinical assessment. All data were de-identified prior to these analyses with data collection, analyses, and de-identification processes under review of the Hospital’s Institutional Review Board; data analyses were deemed exempt from further review per federal guidelines.

### Measures

#### SI

Patients were assessed for thoughts of ending their life (SI) in the past week through the use of the Behavior and Symptom Identification Scale 24 [21]. Patients rated the frequency of these thoughts in response to a single item on a scale from 0 (none of the time) to 4 (all of the time).

#### Demographics

Patients’ age, gender, and ethnicity were obtained from medical records.

#### Diagnoses

Diagnoses were made by the supervising psychiatrist for each patient. Patients could be assigned one to five diagnoses according to ICD-9 diagnostic criteria; while these diagnoses could be for non-psychiatric conditions (e.g., medical conditions of relevance to treatment), patients’ primary diagnoses were exclusively psychiatric in nature. Analyses involving number of diagnoses were conducted solely for psychiatric diagnoses.

#### NSSI urges

Patients completed the Alexian Brothers Urges to Self-Injure Scale [22]. This five-item self-report measure has well-demonstrated convergent and predictive validity, as well as test-retest reliability. This measure has demonstrated high internal consistency and validity in previous studies (Cronbach’s alpha = .92, [22]), and similarly high internal consistency in this sample (Cronbach’s alpha = .93). Each item is rated on a scale from 1 to 7, with total scores ranging from 5 to 35; higher scores indicate greater desire to engage in NSSI.

#### Functions of NSSI

Patients completed the Inventory of Statements About Self-Injury, Short Form [23, 24]. This measure includes 26 items assessing 13 functions of NSSI, each of which is rated on a scale from 0 (not relevant) to 2 (very relevant). Scores are averaged across items for each of the 13 functions, as well as for two overarching factors, interpersonal (social) and intrapersonal (internal) factors. The original (long form) inventory has demonstrated high internal consistency and is appropriately correlated with relevant clinical and contextual measures. The short form has demonstrated nearly identical internal consistency (Cronbach’s alphas range .66 to .80 for 13 subscales) and factor structure as the original form [24]. In this sample, internal consistency was similar to previously published work (Cronbach’s alphas range .67 to .82) with the exception of sensation seeking, which exhibited poor internal consistency (Cronbach’s alpha = .41).

#### NSSI characteristics

Patients completed the Alexian Brothers Assessment of Self-Injury [25], a measure that captures a variety of characteristics about NSSI. Specifically, this measure assesses a variety of specific methods of NSSI in detail; patients were asked how many times they have engaged in each behavior in the past week, on how many days in the past year, how many times per day during the past year, and the age of onset (in years) for each NSSI behavior. Patients were also asked the number of times that they self-injured in the week prior to admission, and to rate the medical severity and impulsivity of their NSSI in the past week. Medical severity of past week NSSI is rated on a scale from 1 (mild, no medical care necessary) to 3 (severe, medical care necessary), while impulsivity of past week NSSI is rated on a scale from 1 (impulsive none of the time) to 4 (impulsive all of the time).

In addition to specific methods of NSSI, patients are asked about their experience of NSSI in several different respects. These items include: desire to stop NSSI, dissociation with NSSI, belief that NSSI is a problem, using substances prior to NSSI, rituals associated with NSSI, feeling more suicidal without NSSI, and engaging in NSSI to avoid being hurt by someone else. Each of these variables is assessed by a single item.

Some patients completed prior versions of the ABASI, resulting in variation in the anchor points for the rating scales on some variables. To standardize responses across different versions of the ABASI, items were recoded using a binary present/absent coding system. For items that were rated on a scale ranging from none of the time to all of the time (NSSI before others hurt you, substances before NSSI, rituals with NSSI), items were coded as present if patients indicated a frequency other than “none of the time.” For items that were rated on a scale ranging from strongly agree to strongly disagree (desire to stop NSSI, dissociation with NSSI, NSSI is a problem, more suicidal without NSSI), items were coded as present if patients indicated agreement or strong agreement and as absent if patients indicated disagreement or strong disagreement. In one version of this scale, the midpoint “unsure” was used; patients who marked “unsure” were coded as missing for that item. For items coded based on frequency of each experience (i.e., specific NSSI methods, NSSI before others hurt you, rituals with NSSI), items were coded as present if patients indicated the experience happened at least once.

Patients were also asked items keyed to proposed diagnostic criteria for NSSI Disorder in DSM-5; these were: experiencing negative thoughts or feelings prior to NSSI, experiencing problems with people before NSSI, experiencing urges to engage in NSSI, and thinking about NSSI. These items were rated on a five-point scale ranging from “none of the time” to “all of the time”. Patients were coded as meeting NSSI Disorder criteria if they reported engaging in NSSI on at least five days in the past year, and rated at least two of the four proposed diagnostic criteria at a frequency of “half of the time” (the midpoint of the scale) or greater.

### Participants

The data presented here were collected from a total of 1520 patients who reported their current (past week) SI at intake to treatment. Patients were predominantly non-Hispanic Caucasian (85.95 %), female (87.70 %), and under the age of 18 (79.80 %). Over 60 % of patients had a primary diagnosis of a mood disorder, with a median of 2 diagnoses out of a maximum of 5. Full sample characteristics can be found in Table 1.

**Table 1:** Sample Demographic and Clinical Characteristics

Variable	*n* (%) or *M* (*SD*)	*M (SD) of SI*
SI	2.24 (1.21)	
Race/Ethnicity		
Non-Hispanic White	1083 (85.95)	2.27 (1.22)
Hispanic	122 (9.68)	2.16 (1.16)
African-American	32 (2.54)	2.19 (1.09)
Asian-American	10 (.79)	1.30 (.95)
Other	6 (.48)	1.83 (.75)
Native American	4 (.32)	3.00 (1.83)
Multi/Biracial	3 (.24)	2.00 (1.73)
Gender		
Female	1333 (87.70)	2.27 (1.22)
Male	187 (12.30)	2.03 (1.14)
Age	16.36 (2.63)	
<18 years old	1213 (79.80)	2.25 (1.20)
18-25 years old	307 (20.20)	2.20 (1.26)
Primary Diagnoses		
Other Mood Disorder	416 (27.44)	1.90 (1.07)
Major Depressive Disorder	390 (25.73)	2.36 (1.18)
Bipolar Disorder	148 (9.76)	2.28 (1.27)
Eating Disorder	15 (.99)	2.53 (1.64)
Anxiety Disorder	31 (2.04)	1.97 (.91)
Substance Use Disorder	6 (.40)	2.50 (1.38)
Other Disorder	507 (33.44)	2.44 (1.27)
Impulse Control Disorder	3 (.20)	1.67 (1.15)
Number of Diagnoses	2.38 (1.16)	

Note: All values are *n* (%) except for age, number of diagnoses, and suicidal ideation (SI), which are *M*(*SD*)

### Data analysis

The clinical outcome assessment program at Alexian Brothers Behavioral Health Hospital was designed to result in a single assessment at each intake to and discharge from the SIRS program. In order to avoid unintentionally assessing treatment effects on suicidality and NSSI characteristics, only data from intake assessments were used. In addition, to prevent over-valuing patients with repeated stays, only the first treatment stay for any given patient was used, regardless of subsequent stays. Due to our interest in further understanding the relationship between NSSI and SI specifically in youth, only data from adolescents (under age 18) and young adults (ages 18 through 25) were analyzed.

Given the risk of increased Type I error from multiple comparisons (total tests of main variables of interest = 54), alpha was corrected from *p* < .01 to *p* < .0046 based on the procedure identified by Benjamini and Hochberg [26]. By controlling false discovery rate (FDR), the Benjamini and Hochberg procedure is less conservative than procedures that control family-wise error, such as the Bonferroni correction. Use of an FDR control method with a more stringent starting alpha balances the need to avoid inflated Type I error while also avoiding an extreme reduction in power for these exploratory analyses.

To evaluate the relationship between SI (an interval scale variable) and binary variables (for example, presence of a specific NSSI method), independent-samples t-tests were used. To evaluate the relationship between SI and other interval variables (for example, number of methods of NSSI in the past year), Pearson’s correlation coefficients were used. In order to provide a common metric in which to evaluate the relative importance of NSSI characteristics to SI, all effects are reported as Cohen’s *d*s, a standardized measure of effect size [27]. This measure provides an estimate of the magnitude of the difference between two groups, where an effect size of .2 is considered “small,” .5 is considered “medium,” and .8 is considered “large” [27]. Reported values characterize the relationship between SI measured dimensionally and our constructs of interest; analyses were repeated with SI measured as a binary variable (presence or absence of any SI in the past week), and results were almost identical and followed the same pattern as those presented here.

## Results

### SI and Demographic Characteristics

A plurality of patients reported no SI over the week prior to intake (34.28 %), while a further 31.18 % reported thinking about suicide a little of the time, 16.25 % reported thinking about suicide half the time, 12.57 % reported thinking about suicide most of the time, and 5.72 % reported thinking about suicide all of the time. The mean level of SI was 2.24 (median = 2), a score between “a little” and “half” the time.

There were no statistically significant differences in mean SI level by ethnic group (Cohen’s *d* = .12, *p* = .13, higher SI in non-Hispanic Caucasians compared to other ethnic groups), gender (Cohen’s *d* = .20, *p* = .008, higher SI in females compared to males), age (Cohen’s *d* = .04, *p* = .51, higher SI in adolescents compared to young adults), or number of psychiatric diagnoses (Cohen’s *d* = −.06, *p* = .26, lower SI associated with more psychiatric diagnoses). Patients with a primary diagnosis of a mood disorder exhibited significantly less SI than patients with any other primary Axis I diagnosis (Cohen’s *d* = .22, *p* < .001).

### NSSI Behaviors

For these analyses, patients were compared on past week and past year measures of NSSI behaviors. Higher endorsement of SI in the past week was significantly associated with medical severity of NSSI in the past week, as well as with greater urge to engage in NSSI in the past week. SI was not associated with NSSI frequency in the past week, nor with impulsivity of NSSI behaviors. Full results are reported in Table 2.

**Table 2:** Comparisons on Past Week and Past Year NSSI Methods and Frequency

Past Week NSSI Measures	*n*	*d*	*p*
Number of NSSI episodes (ABASI)	1514	.13	.01
Medical severity of NSSI (ABASI)	1239	.29	<.001
Impulsivity of NSSI (ABASI)	1234	-.01	.87
Craving for NSSI (ABUSI total score)	1476	1.16	<.001
Past Year NSSI Methods (ABASI)			
Cut	676	.13	.09
Scratch	676	.20	.01
Burn	676	.12	.11
Tattoo/Pierce	676	.01	.91
Choke	676	.33	<.001
Pull Hair	676	.20	.01
Draw Blood	676	.09	.25
Insert/Embed	676	.19	.01
Hit	345	.45	<.001
Bang	676	.28	<.001
Prevent Wound Healing	676	.24	.002
Fall Down	676	.00	.99
Break Bones	676	.11	.16
Gouge	676	.05	.51
Fight	676	.11	.14
Ingest/Swallow	676	.09	.25
Mutilate Genitals	343	.00	1
Worsen Medical Condition/Ignore Medical Advice	676	.23	.003
Past Year Number of NSSI Methods (ABASI)	676	.52	<.001
Past Year Number of Days with NSSI (ABASI)	345	.65	<.001

SI was not significantly associated with 5 of the 18 specific methods assessed in the preceding year. Of those methods that were significantly associated with SI, most were what are often considered “minor” NSSI (hitting, banging, preventing wounds from healing) [28]. Other methods that were associated with higher SI included choking and intentionally worsening a medical condition or intentionally ignoring medical advice (see Table 2 for more detailed information). Interestingly, more severe or unusual methods of NSSI (e.g., burning, breaking bones, or swallowing dangerous substances) were not associated with SI, suggesting that indices of NSSI severity may not necessarily be indices of SI severity.

The total number of NSSI methods used in the past year was significantly correlated with current SI. While no true value for past year NSSI frequency was available, patients reported the number of days on which they engaged in each of the 18 methods; the rank-transformation of the sum of these values (or a rough approximation of NSSI frequency) was significantly correlated with current SI.

### NSSI Functions

All but four of the 13 ISAS-SF functions were significantly positively associated with SI in the past week; the only functions not associated with SI were interpersonal functions (self-care, peer bonding, interpersonal influence, revenge, full details in Table 3). Of the functions associated with SI, the strongest relationships were between recent SI and anti-suicide and self-punishment NSSI functions. Both the ISAS-SF intrapersonal and interpersonal scales were significantly associated with SI, although the effect size was larger for intrapersonal than interpersonal functions.

**Table 3:** Comparisons on NSSI Functions

ISAS-SF Scales	*n*	*d*	*p*
Affect Regulation	1510	.46	<.001
Interpersonal Boundaries	1514	.35	<.001
Self Punishment	1507	.72	<.001
Self Care	1514	.14	.01
Anti-Dissociation	1515	.56	<.001
Sensation Seeking	1514	.23	<.001
Anti-Suicide	1513	.72	<.001
Peer Bonding	1512	-.04	.41
Interpersonal Influence	1511	.14	.01
Toughness	1509	.34	<.001
Marking Distress	1513	.43	<.001
Revenge	1513	.13	.01
Autonomy	1469	.29	<.001
ISAS-SF Intrapersonal	1516	.88	<.001
ISAS-SF Interpersonal	1516	.34	<.001

### Other NSSI Characteristics

Patients with greater current SI at intake were more likely to report feeling more suicidal without NSSI. There was also a relationship between current SI and dissociation during NSSI, as well as patients engaging in NSSI before someone else could hurt them. Patients who met proposed diagnostic criteria for NSSI disorder exhibited significantly greater SI than those who did not. Other characteristics of NSSI behavior, including rituals with NSSI, use of substances during or before NSSI, and age of NSSI onset were not associated with current SI. There was a significant relationship between current SI and the belief that the patient’s NSSI was a problem; however, there was no relationship between current SI and the desire to stop or decrease NSSI. Detailed results regarding these NSSI characteristics can be found in Table 4.

**Table 4:** Comparisons on NSSI Characteristics from the Alexian Brothers Assessment of Self-Injury (ABASI)

Variable	*n*	*d*	*p*
Engage in NSSI before being hurt	676	.25	.001
Engage in rituals with NSSI	676	.11	.14
Dissociate during NSSI	572	.37	<.001
Become more suicidal without NSSI	526	.98	<.001
NSSI is a problem for me	633	.35	<.001
I want to stop or decrease NSSI	624	.05	.54
Met DSM-V NSSI disorder criteria	345	.54	<.001
Use drugs before/during NSSI	345	.07	.54
Age of NSSI onset	662	-.08	.28

## Conclusions

This study sought to clarify the relationship between NSSI and suicidality by examining how characteristics of NSSI were associated with SI. Given the strong relationship between suicidal thoughts and behaviors [29], understanding how NSSI is associated with SI is critical to further understanding the role of NSSI in behaviors such as attempted and completed suicide.

The most robust correlates of recent SI were those associated with NSSI serving a strong intrapersonal regulatory function, in particular, to avoid suicide; these correlates included greater NSSI craving in the past week, becoming more suicidal without engaging in NSSI, and using NSSI to avoid suicide. SI was also strongly correlated with the overall intrapersonal functions of NSSI, to a greater extent than interpersonal functions.

These results suggest several potential pathways to better understand the specific associations between NSSI and SI. It is possible that exposure to stressful or difficult life experiences acts as a third variable, contributing not only to a greater desire to use NSSI to cope with stressors, but also to a greater concurrent desire to escape those stressors through suicide. Another potential pathway is that NSSI occurs first – to regulate internal states – but suicidality increases when NSSI fails to address underlying emotional pain. Finally, self-injurers who experience suicidal thoughts may subsequently notice that NSSI helps ameliorate these thoughts in the short term, leading to a greater use of NSSI for this and other intrapersonal functions. Regardless of the potential causal pathway through which these characteristics are related, these results provide valuable evidence of the importance of a strong functional assessment of NSSI to better understand suicide risk among individuals who self-injure.

Several behavioral measures of NSSI severity in the past year were associated with current SI, including number of days on which NSSI was used in the past year and number of NSSI methods, which is consistent with research suggesting that higher NSSI frequency and number of methods may be associated with greater risk of suicidal behavior [19]. Interestingly, these measures were more strongly associated with recent SI than the measures of NSSI severity (frequency and self-rated medical severity) in the past week, during the same period as the SI. These results suggest an important caveat for clinicians conducting risk assessment with people who self-injure, namely, that individuals with infrequent or medically minor current NSSI may still be at elevated risk of SI by nature of their more distal NSSI history.

With respect to NSSI methods, current SI was more strongly associated with what are often referred to as “minor” NSSI, such as interference with wound healing, than more severe forms of NSSI, such as breaking bones. This was the case for more unusual forms of NSSI, like mutilating genitals, but was also the case for more common forms of severe NSSI, such as cutting or burning, suggesting these findings are not simply due to a low base rate of extreme behaviors. This finding is in contrast with the result, earlier described, suggesting that recent NSSI of higher medical severity is, to some extent, associated with higher recent SI. There are several possible explanations for these findings. First, we considered whether this relationship might be artificially inflated by the inclusion of patients admitted to the SIRS program to treat serious SI who engaged in little to no NSSI. To test this, we repeated our analyses using only the subset of patients who met DSM-V proposed criteria for NSSI Disorder; in this case, no methods were significantly associated with current SI, although the general pattern of larger relationships between suicidality and “minor” NSSI methods remained, suggesting that inclusion criteria for the SIRS program cannot fully explain these results. Another possibility is that less severe forms of NSSI may be done more habitually than more severe NSSI, and that this indexes greater frequency or duration of negative affect, which could be associated with SI. It may also be that low medical severity NSSI is less likely to be noticed by others and, hence, more strongly associated with intrapersonal (rather than interpersonal) NSSI functions, which were also associated with SI in this sample. Further research with more detailed measures of NSSI methods (e.g., frequency and duration of each method used) will be useful in clarifying this relationship.

Additional findings indicate that individuals who self-injure and report elevated levels of SI exhibit more clinically severe NSSI; for example, patients who met proposed DSM-5 diagnostic criteria for NSSI Disorder reported significantly greater SI. This was in contrast to other characteristics that have often been considered to index NSSI severity, for example, using drugs or alcohol before or during NSSI, overall NSSI frequency, or engaging in NSSI rituals. These results suggest that the proposed DSM-5 criteria appropriately identify clinically significant NSSI [30], and supports their use in place of other rough proxies of NSSI severity. SI was associated with reporting that NSSI was problematic, but was not associated with a desire to stop NSSI, suggesting that self-injurers experiencing concurrent suicidality may not be more or less amenable to treatment than their nonsuicidal counterparts. This could be due, in part, to the reported use of NSSI as a way to avoid suicide, and the likely resistance on the part of some patients to give up what they perceive to be an important coping mechanism. This finding may prove useful to clinicians faced with self-injuring and suicidal patients for whom motivation and treatment adherence are often limited.

As is the case in any research, this study suffers from several limitations. First, data were collected from a sample of adolescents and young adults receiving treatment for NSSI in an acute treatment program; as such, this population exhibits quite clinically significant NSSI, and these findings may not be applicable to other populations of individuals who self-injure, for example, community populations of adolescents in settings such as schools. For example, previously published research using a sample of college students found no relationship between intrapersonal functions of NSSI and SI [20], which is in contrast to our findings presented here. Second, all characteristics associated with NSSI and SI were assessed using self-report measures, such that the responses may have been subject to a variety of recall and reporting biases. While most of these characteristics require self-reported assessment, research using more thorough structured interviews to assess NSSI and SI might yield different results. Third, all measures were cross-sectional in nature, which prevents us from making causal inferences about the relationship between NSSI and SI; further longitudinal work is needed to understand whether these aspects of NSSI are actually predictive of later SI, particularly in light of research suggesting that cross-sectional correlates of NSSI may not prospectively predict NSSI behaviors [31]. Fourth, due to concerns about patient burden when completing assessments, many of our variables of interest were indexed by a single item; future research focusing on understanding how NSSI characteristics are associated with SI will benefit from the use of more extensive, psychometrically-validated measures of these constructs. Finally, while our analyses yielded a range of effect sizes depending on the variable being evaluated (Cohen’s *d*s ranged from -.08 to 1.16), most analyses yielded results of small effect (e.g., Cohen’s *d*s at or below .3). This suggests that, while some NSSI characteristics may provide valuable information to understand SI in this population, there remains extensive variability in SI that cannot be explained by NSSI alone; as such, evaluation of other known risk factors for SI (e.g., depression, hopelessness, substance use) should continue to be a part of clinical assessment of individuals engaging in NSSI who may also be at risk for SI.

These findings suggest important areas of consideration for clinicians conducting suicide risk assessment with self-injuring clients. Given the strong relationship between NSSI and suicidal thoughts and behaviors, and the association between SI and later suicidal behaviors, understanding what factors are associated with SI in this population is crucial. In particular, understanding the NSSI characteristics that are associated with SI provides unique insight into self-injurers specifically, rather than trying to assess only more general risk factors for SI (e.g., hopelessness, perceived burdensomeness). Future research should continue to investigate how NSSI characteristics are associated not only with SI, but also with attempted and completed suicide, in order to fully understand the complex relationship between nonsuicidal and suicidal self-injury.

## Abbreviations

NSSI: Nonsuicidal self-injury
SI: Suicidal ideation
SIRS: Center for Self-Injury Recovery Services Program
